# INTO THE VOID: A CAREER IN INCONTINENCE… AND BEYOND

**DOI:** 10.1093/geroni/igac059.837

**Published:** 2022-12-20

**Authors:** Neil Resnick

**Affiliations:** University of Pittsburgh, Pittsburgh, Pennsylvania, United States

## Abstract

Long-believed inevitable with age, urinary incontinence (UI) --like dementia – has proved neither ineluctable nor untreatable. Moreover, older adults remain continent despite abnormal lower urinary tract (LUT) function! The key is that the LUT is just one risk factor, and incontinence results from contributors at every level: from drugs and diseases beyond the LUT, to problems of brain and physical function, to multiple issues within the LUT itself. Such insights have provided the rationale for a nontraditional diagnostic and therapeutic strategy, one that has not only improved outcomes but also enabled creation of previously-believed heretical approaches that address the LUT last and only if necessary. In addition, this approach enabled development of Medicare’s Minimum Data Set for frail elderly and, more importantly, may facilitate development of scalable ways to raise the floor of care for other geriatric syndromes as well.

